# The key components of a successful model of midwifery-led continuity of carer, without continuity at birth: findings from a qualitative implementation evaluation

**DOI:** 10.1186/s12884-021-03671-2

**Published:** 2021-03-12

**Authors:** N. Dharni, H. Essex, M. J. Bryant, A. Cronin de Chavez, K. Willan, D. Farrar, T. Bywater, J. Dickerson, on behalf of the Better Start Bradford Innovation Hub

**Affiliations:** 1 Royal College of Obstetricians & Gynaecologists, London, UK; 2 Better Start Bradford Innovation Hub, Bradford Institute for Health Research, Bradford, York, UK; 3 Department of Health Sciences, University of York, York, UK; 4 Leeds Teaching Hospitals NHS Trust, Leeds, UK; 5 Nuffield Centre for International Health and Development, University of Leeds, Leeds, UK; 6 Maternal and Infant Health, Bradford Institute for Health Research, Bradford, UK

**Keywords:** Midwifery, Continuity of carer, Pregnancy, Qualitative, Implementation

## Abstract

**Background:**

Recent UK maternity policy changes recommend that a named midwife supports women throughout their pregnancy, birth and postnatal care. Whilst many studies report high levels of satisfaction amongst women receiving, and midwives providing, this level of continuity of carer, there are concerns some midwives may experience burnout and stress. In this study, we present a qualitative evaluation of the implementation of a midwife-led continuity of carer model that excluded continuity of carer at the birth.

**Methods:**

Underpinned by the Conceptual Model for Implementation Fidelity, our evaluation explored the implementation, fidelity, reach and satisfaction of the continuity of carer model. Semi-structured interviews were undertaken with midwives (*n* = 7) and women (*n* = 15) from continuity of carer team. To enable comparisons between care approaches, midwives (*n* = 7) and women (*n* = 10) from standard approach teams were also interviewed. Interviews were recorded, transcribed and analysed using thematic analysis.

**Results:**

For continuity of carer team midwives, manageable caseloads, extended appointment times, increased team stability, and flexible working patterns facilitated both care provided and midwives’ job satisfaction. Both continuity of carer and standard approach midwives reported challenges in providing postnatal continuity given the unpredictable timing of labour and birth. Time constraints, inadequate staffing and lack of administrative support were reported as additional barriers to implementing continuity of carer within standard approach teams. Women reported continuity was integral to building trust with midwives, encouraged them to disclose mental health issues and increased their confidence in making birth choices.

**Conclusions:**

Our evaluation highlighted the successful implementation of a continuity of carer model for ante and postnatal care. Despite exclusion of the birth element in the model, both women and midwives expressed high levels of satisfaction in comparison to women and midwives within the standard approach. Implementation successes were largely due to structural and resource factors, particularly the combination of additional time and smaller caseloads of women. However, these resources are not widely available within the resources of maternity unit budgets. Future research should further explore whether a continuity of carer model focusing on antenatal and postnatal care delivery is a feasible and sustainable model of care for all women.

**Supplementary Information:**

The online version contains supplementary material available at 10.1186/s12884-021-03671-2.

## Background

There is evidence indicating that a midwife-led continuity of carer (CoC) approach for women with low risk pregnancies can reduce the risk of intervention during labour and increase women’s satisfaction with their maternity experience [1, 2]. Midwife-led continuity of carer can be achieved by providing a named midwife to each mother, who follows women throughout their pregnancy, birth and postnatal period. In 2016, the national maternity review, ‘Better Births’, recommended that improved quality and safety of maternity services in England could be attained by providing CoC to every woman throughout pregnancy, birth and postnatally [3]. Several studies have reported high levels of midwives’ satisfaction with continuity working where factors such as professional autonomy, providing relational care and forming positive relationships with women have been cited as being central to increased job satisfaction [4–8]. However, there are concerns about aspects of the midwife led CoC across the antenatal, intrapartum and postnatal periods that may have a negative impact on midwives due to increased out of working hours on-call, inadequate staffing levels and difficulties with work-life balance [5, 9–11]. Unless we have a workforce that is willing and able to work in this manner, the longer-term implementation of CoC models remains a challenge. With increased strategic and policy directives focusing on providing midwife led CoC, it is important to understand how to successfully implement and sustain CoC models.

Prior to the Better Births review, and recognising the challenges in delivering full CoC, Better Start Bradford (a Big Lottery Community Fund initiative, see www.betterstartbradford.org) commissioned Bradford Teaching Hospitals NHS Foundation Trust to implement a midwife-led CoC model. To enhance relational care and prevent midwifery burnout, the model required continuity of carer for the antenatal and postnatal periods and did not require midwives to be present at birth.

### CoC model applied in Bradford

Six midwives and a team leader were employed to deliver a CoC model wherein model women saw their named midwife or ‘back-up buddy’ for their routine antenatal and postnatal care, but not during labour and birth. CoC was defined as the proportion of women’s appointments with their named midwife or back-up buddy. In this model, 94% of women received CoC during the antenatal period, compared to 65% in usual care, and 70% of women received CoC in the postnatal period (no comparable data was available for postnatal continuity in usual care) [12].

The service was available to pregnant women who were registered with five general practices across three inner city areas and aimed to support approximately 500 women per year between October 2015 – October 2018. As well as providing relational care, the model also aimed to increase women’s engagement with antenatal care and early detection of problems during the perinatal period, in particular mental health; improve women’s satisfaction and sense of empowerment during pregnancy and after birth; improve women’s nutrition and health during pregnancy and reduce behaviours such as smoking. Table 1 summarises the key differences in the delivery of care between the CoC and usual care approaches.

**Table 1:** Comparison of the CoC model and standard team approach

CoC model	Standard team approach
Women receive care from the same named midwife or a ‘back-up buddy’ throughout pregnancy and after birth	Women to see a midwife from their team without an emphasis on continuity of carer.
Each midwife will have a maximum caseload of 60 women at any one time.	Caseloads of around 100–120 women per midwife.
An additional home visit offered prior to the booking appointment to introduce the service and offer initial pregnancy health advice	No home visit available.
90 min for booking appointments and 30 min for antenatal follow up appointments	60 min for booking appointment and 20 min for antenatal follow up appointments.
Midwives act as a care coordinator for women with higher risk pregnancies, consultant led or other hospital care. May also attend hospital appointments with women where desired/required.	Standard care from midwives covering the community service, no availability to attend hospital appointments.
Additional postnatal appointments offered if required, tailored to an individual woman’s level of need. All postnatal care at home.	Same, but the ability to tailor care or to offer extra appointments may be more restricted. Limited home visits postnatally.

### Evaluation aims and objectives

In this paper we present a qualitative evaluation of the implementation of the model exploring the key components of a successful model of CoC without continuity at birth, specifically: whether the model was implemented as planned (fidelity); what barriers and facilitators affected the delivery of the model; how satisfied midwives and women were with the CoC model (and how this compared with the satisfaction of midwives and women from the standard approach); and how feasible the wider implementation model would be including a consideration of contextual factors.

## Methods

### Study design and setting

This was a qualitative study using an implementation evaluation framework [13] underpinned by the ‘conceptual framework for implementation fidelity’ [14, 15]. The CoC model was implemented in an ethnically diverse and socio-economically deprived inner city areas of Bradford as a part of the Better Start Bradford initiative [16]. The study was undertaken as part of a wider evaluation of the Better Start Bradford early intervention programme through a novel experimental birth cohort (Born in Bradford’s Better Start, BiBBS) that recruits pregnant women living in these areas [16].

### Participant recruitment

#### Midwives

Midwives working in the CoC team and midwives working within a standard team approach within the same geographical area, and caring for women with similar health and social needs, were approached for interviews by clinical midwifery managers, who were also invited to participate in the study. Consenting participants were then contacted by a member of the research team to arrange a convenient time for face to face interviews, or telephone interviews where preferred.

#### Women

Study invitation letters and information sheets were mailed to a sub-sample of women who had already consented to be contacted as part of the experimental BiBBS cohort study [16]. We included women who did not speak English either by interviewing them in their home language or with the assistance of interpreters. Women were invited to take part between three- and six-months post birth to ensure more accurate recollection and reflection on experiences of care. Participation was subsequently confirmed with women via telephone calls 2 weeks after the postal study invitation. Prior to starting interviews, written informed consent was obtained from all participants. Participants were reminded their data would be anonymous, stored securely and that they could withdraw at any time.

The BiBBS study received ethical approval by Bradford Leeds NHS Research Ethics Committee (15/YH/0455), and research governance approval from Bradford Teaching Hospitals NHS Foundation Trust. This additional evaluation was deemed as service evaluation and as such did not require formal ethical review by the NHS or any other research ethics committee (HRA decision 60/88/81; please see supplementary information for confirmation of this decision). Nonetheless, the principles of good clinical practice and research governance were upheld through the provision of information sheets, signed informed consent and data protection and confidentiality as per the evaluation protocol [13].

### Data collection

#### Interviews

All interviews were conducted in person, digitally recorded and transcribed verbatim. Interviews with women took place in their homes (by ND & AC) and interviews with midwives were undertaken (by ND & HE) in a private room at their base location.

#### Development of topic guides

Topic guides were based on the Theoretical Domains Framework (TDF [17, 18]; which encompasses a comprehensive range of constructs from theories of behaviour change including emotions, beliefs about capabilities, knowledge, skills and social influences. Whilst the interview questions for midwives and mothers differed, use of the TDF ensured the underlying theoretical concepts explored in both interviews were the same. The topic guides for midwives, midwifery managers and women are included as supplementary information. Pilot interviews were conducted with two women and two midwives to gain feedback on the length of the interview topic guides, establish the suitability of the questions, how well they were understood, and if any questions should be removed or added. No changes to the topic guides were required after piloting.

### Analysis

Data were coded using a hybrid process of both inductive and deductive thematic analysis. Thematic analysis (TA) is a widely used method in evaluative studies which seeks and reports patterns inherent within the data [19]. Themes were coded according to the conceptual framework for implementation fidelity’ [14, 15] including: fidelity, reach, participant responsiveness and satisfaction, and strategies to facilitate implementation. Given the high levels of ethnic and socioeconomic diversity in our study area, we explored patterning of themes by individuals’ ethnicity, socioeconomic circumstances and English language ability. Deviant cases; where participants’ responses appeared to contradict patterns emerging from the data were also discussed. The familiarisation stage of analysis began whilst interviews were being undertaken to allow the formation of the thematic framework and monitoring of data saturation.

Transcripts were coded systematically and iteratively until the analysis team were satisfied that the analysis framework adequately captured the data and saturation had been achieved. Initially, two researchers (ND & AC) coded the same six transcripts and met to compare the emerging coding framework. Once the framework was agreed, the researchers continued independently coding the remaining transcripts with regular consensus meetings. Data were managed within the Nvivo data management programme (NVivo qualitative data analysis Software; QSR International Pty Ltd).

## Results

### Participants

#### Midwives

Fourteen community midwives, all female, working in a similar geographical area participated in interviews. Interviews included six midwives from the CoC team, five midwives from standard approach teams and three team leaders across both models of care. Midwives had been qualified for an average of 12.2 years (range 1–33 years).

#### Women

Twenty-five women from our ongoing BiBBS cohort study [16] participated in interviews of which 15 women received care from the CoC team and 10 women received care from the standard approach team. Participants were representative of the Better Start Bradford area with the majority of participants (*n* = 20) of South Asian origin and the remainder from a diverse range of ethnic backgrounds. Women were aged between 20 and 40 years and just over half (*n* = 13) were first time mothers. Most participants were interviewed in English (*n* = 19), five participants were interviewed by a bilingual researcher (ND) and one participant was interviewed with the assistance of an interpreter.

### Findings

#### Fidelity: midwives’ perspectives of fidelity of the model in practice

During qualitative interviews, midwives in the CoC team reflected on their experiences of working in the CoC model and the value of having the time to establish and maintain contact to benefit women as well as their own professional practice. There were also examples of CoC being repeated for women returning to the team for subsequent pregnancies with referrals being allocated to their original midwife.

*OK, so the key components here are having more time and less women to care for so you can get to know each individual woman a bit better, they’re not just a blank name until we walk in a room and then you recognise them. And I do think we build up much better relationships with the women and the families, especially now I’m starting to get through some women who have had their first pregnancy with me and are now on to their second pregnancies (CoC Midwife, 4)*

Continuity for antenatal appointments was deemed easier to implement as midwives had the autonomy to schedule appointments according to their working patterns and scheduled leave, although was at times still challenging due to sickness and staff changes within the team during the early phases of implementation, which impacted overall levels of continuity provided to women. Continuity for postnatal appointments was reported to be more challenging; for example, for women who delivered over a weekend, as some postnatal visits are required to take place at set time points (e.g. day 1 after birth).

*The intention is that women see their named midwife or buddy 90% of the time, which in practice generally works quite well, certainly for me my buddy works very well but there has been sickness in the team which has made it more difficult and a change of midwives that’s made it more difficult. Postnatally it’s affected by the fact that postnatal care in Bradford in particular is very prescriptive as to what days women are visited, so if it’s a woman has to be seen on a given day and neither their midwife nor buddy are working then they still have to have a midwife visit and it will be somebody else out of the team. (CoC Midwife, 5)*

### Reach: what were the characteristics of women supported by the CoC team?

Midwives across the CoC and standard approach teams reported similarly high levels of vulnerability and safeguarding concerns in the women they were caring for. Regardless of experience, several midwives across both teams reported they were working with a complex caseload of women for the first time. Key challenges and vulnerabilities reported by midwives included caring for a highly transient population, language barriers, women’s health complications, domestic violence, female genital mutilation (FGM), mental health and cases of extreme poverty.

*I've definitely got a real range of different ethnic minority groups with different languages from all over the world really … I've got a lot, and it seems to be increasing as well, a lot of obese, raised BMI ladies...I've had a lot of ladies with FGM and then we've got to do the safeguarding and follow the FGM guideline pathway so that’s been quite a new thing for me, even though I've been a midwife since 1990, I've not come across that before in other caseloads that I've worked and that’s quite new I suppose to the area and the recording of all of that. Safeguarding, mental health … (CoC midwife, 7)*

*The caseload I have is quite a complex one … and they tend to be very transient in coming and going, they may be here one week and then they’ve disappeared the next time you’re supposed to have seen them (Standard approach midwife, 11)*

Whilst the complexity of women’s needs were shared across teams, midwives from the CoC team were less pressured and constrained by time to support their women compared to midwives in standard approach teams. Furthermore, the focus on continuity and building relationships with women appeared to facilitate women’s continued attendance for their antenatal appointments as well as increased acceptance of support offered. Where CoC midwives reported sufficient time to address women’s concerns, many standard approach midwives conversely reported spending additional time outside of their working hours to complete referral paperwork for their women.

*I’ve got sufficient time in the office mostly to ring Social Services or refer women to wherever they need to be referred to and I’m not thinking, you know, please don’t tell me something’s majorly wrong because I’ve only got 5 minutes, I don’t have that pressure and I think a lot of community midwives probably still do and that’s the harsh reality of it. (CoC Midwife, 2)*

*… every clinic there’ll be somebody with a problem where you’ve got to do then do something for them, do a mental health referral, do a safeguarding referral, refer them to the GP or something, and so we do rely on women that actually miss appointments, which is really sad. I think at the moment the way we are a lot of the midwives actually work overtime that’s unpaid and that’s how we get around it, so mums who have, or midwives that have got children who can’t stand behind or can’t come early in the morning they struggle as well keeping on top of the caseload because I think a lot of it is on goodwill from the midwives part because we do spend so much overtime sorting out paperwork. (Standard approach midwife, 9)*

### Participant responsiveness and satisfaction

All CoC team midwives reported high levels of job satisfaction, reduced stress and increased perceptions of role fulfilment which they deemed to be a result of working in the CoC model. Moreover, CoC midwives felt they had sufficient time to support women, build trusting relationships and provide good quality antenatal and postnatal care. Several CoC team midwives also reflected on their previous experiences of working within a standard approach where time and resource constraints appeared to affect perceptions of the quality of care provided to women as well as their own job satisfaction.

*Um, when I was working in the other community teams you were on, constantly watching the clock all the time, whereas now you don’t have to, I mean you do because obviously you’ve got another woman to see, but you’re not stuck with that like something right over your shoulder thinking, oh I’ve got to hurry up, I’ve got to hurry up, I can’t talk to you because I’ve got another lady coming. (CoC Midwife, 3)*

*I think I make a difference, I like my job whereas before, running around like an idiot … I just feel as if I'm doing what I trained to do now and doing a better job (CoC Midwife, 5)*

Accounts of both standard approach midwives and CoC midwives who previously delivered the standard approach evoked a sense of tension between time and resource constraints, and the desire to provide good quality, personalised care to women. Many standard approach midwives reported feelings of stress and burnout due to time pressures and a lack of role fulfilment.

*Stressed and that’s only because I haven’t got the time to do what I want to do … And feeling that you’re being rushed into things and rushing in and out because you’ve got so much to do in a set time and you can’t get it all done. (Standard approach midwife 12)*

For women who received their care from the CoC team, high levels of satisfaction were reported and associated with increased reassurance and confidence with their pregnancy and birth choices. In addition, women felt there was sufficient time to ask questions and reported receiving advice and support on a wide range of issues including fears about labour, help with breastfeeding, post labour recovery and for one woman, advice on introducing a new baby to an older autistic sibling.

*The midwife told me everything and I then made the decisions myself. She didn’t say do this and do that. I told her myself, for example can I try for a normal delivery, and she said of course you can try. There are many women that try for a normal delivery and end up having an operation. I wrote it down that if I give up during normal delivery you can do an operation. (Mother 3, CoC, Urdu speaker)*

Women receiving CoC also valued the increased accessibility and flexibility of their midwives in terms of scheduling appointments, home visits during pregnancy where requested and all postnatal care given at home as well as having a direct office line that midwives could be contacted on at any time.

*I don’t know if this was available to anyone, there’s always someone there for you, if they’re going on holiday, they’d always let you know, leave numbers and everything so yeah, that was something good as well. You always know, whether you’ve got any problems, you just ring up and they’ll advise you to see a GP or anything like that. (Mother 1, CoC)*

Several women compared satisfaction with care received by the CoC team to care they had received during previous pregnancies, with many women reporting higher levels of satisfaction with information, care and particularly emotional support provided by CoC midwives. Women reported being more informed and reassured about both their own health and the health of their baby during pregnancy.

*I found it really well, it was better than what I got treated before, it was very good... She told me everything what was happening with my baby, what’s going to happen in the next four weeks, what am I meant to be feeling and what it’s not safe to be and if I have any worries to contact her and stuff. She kept telling me that and that reassured me that I know she’ll be there to help me when something happens or if anything goes wrong, she’ll be there. Last time, when I went to her, “Everything’s normal, everything’s fine, you can go. If you’re feeling a bit emotional about stuff, that’s fine.” And, as I say, she just used to send me home, “It’s all fine,” like within 10 minutes, to go in and get all the paperwork done and get out. (Mother 11, CoC)*

The relational aspect of care and focus on women’s needs appeared to be integral for women to build trust with their midwives and encouraged the disclosure, and acceptance of support for any concerns women had. Both women and midwives highlighted women’s mental health as a focal area of discussion for appointments with the CoC team.

*Well, every appointment that I had, the first question was, “How are you feeling? Is there anything that you’re concerned about?” So, you know, despite anything else, and looking at anything else, it was always about my health, and how I feel, and how, you know, how I felt with the baby, which, which I found quite good, as well, and it was a thing of, even though, maybe, my appointments had weeks’ gaps, it was, if I needed any help at any time, I could call and leave a voicemail (Mother 10, CoC).*

This was in contrast to the experiences of women who had received care within a standard approach, where many reported feeling rushed during their appointments resulting in some women not disclosing any mental difficulties they had been experiencing. Many women receiving care within the standard approach advocated for longer appointments to allow them to discuss all their concerns, particularly regarding mental health during appointments.

*So that kind of makes me feel that, even if I say that I have a concern, that, even if it seems like she's attending to it, she doesn't actually, or she forgets or something like that, you know what happens. It's just, it feels they're very busy, that's how it feels, so I can't blame them for that. (Mother 20, Standard approach)*

*Well with them not really because I never told them, you know, my story, they literally, when I suggest go they said to do the same thing, get the … measure my belly, get the blood test, that’s all, and asking if there’s anything new, that’s all, I wasn’t really going with them with the details, yeah. (Mother 22, Standard approach)*

Although women cared by the CoC team did not always see their named midwife, many felt there was good communication between midwives in the team and, instances where they were unable to meet with their CoC midwife, reported receiving similarly high levels of care from other midwives. Furthermore, for women who had experienced the standard approach with previous pregnancies, the extended appointment times and level of attention they felt they received from the CoC team outweighed their preferences for seeing the same midwife.

*I didn’t actually see my same midwife because she went on holiday but she’d always let me know that she wouldn’t be here and somebody else would be here for my, after my birth, you know, the baby, she let me know. It was similar help, you know, we’re talking to my midwife and a lot of them start getting so used to the one they’ve been seeing all through and then they’ve got someone new. But as long as they can sort of work out or work the same way and provide the same help, I don’t think it should be any problem. (Mother 1, CoC)*

### What strategies were employed to facilitate implementation of the model?

During interviews, midwives from the CoC team suggested that the resources and structure of the CoC model allowed them to practice in a different capacity than they had previously experienced. Implementation successes were largely attributed to reduced caseload sizes, extended appointment times (allowing midwives to build relationships with women); increased flexibility and autonomous working and diary management; and the stable and fixed nature of the team. All of these elements facilitated the delivery of personalised care for women and enhanced midwives’ satisfaction.

#### Caseloads

CoC midwives had an average caseload size of 55 women, that being the number of women being cared for at any one time. This compared to caseloads of 120–140 women reported by standard approach midwives. CoC midwives reported the reduced volume of women allowed them to provide more personalised and relational care for women, which was helpful in identifying concerns and areas of support for women early.

*I think it's all about the relationship building, but also about your care itself as well, so if you've got concerns with a particular lady or the growth of the baby or mental health issues, or anything like that, you know that woman, you know what the issues are as soon as she comes to you, and because we've got smaller caseloads we're more able to keep on top of that as well, rather than having hundreds of women where we can't remember who's who and which, who's got what complications (CoC midwife, 1)*

#### Extended appointment times

Extended appointment times for the CoC team allowed midwives more time with women to answer questions, provide detailed information enabling informed choices for women. Women, particularly those for whom English was not their first language, also valued the additional time with midwives in terms of the information and support they were given compared to their previous pregnancies.

*This pregnancy I felt they explained things more, like you need to do this and do that. The way they spoke was very good. If I didn’t understand they would explain it 2 or 3 times. There was no need to call an interpreter; they spoke so clearly and well. (Mother, Urdu speaker, CoC)*

For CoC team midwives, extended appointment times were deemed sufficient given the complexity of the women and their surrounding situational factors. Moreover, the combination of additional time, reduced caseload sizes and familiarity with women allowed midwives to truly provide enhanced and personalised care whilst building relationships and holistically supporting women’s clinical, psychological and social needs. Once again, CoC midwives distinguished their current experiences and ways of working to their previous practice, with particular emphasis on the lack of time in the standard approach to probe women’s wider concerns or knowing critical information about women when contacted by social care and other safeguarding agencies.

*… when we’ve been community midwives before we might have had 15 or 20 minutes to see women and … it’s really probably awful to say but almost like a bit like a tick box exercise because you’ve got to have them in and out and there’s just so many women coming at once and sometimes it’s really hard to get to the bottom of what they need whereas with the personalised midwifery, we have that extra 10 minutes to spend with the women each antenatal appointment and it does give us sufficient time to, sometimes I still run out of time now, talk about the breastfeeding and the birth plan, assessing their mental health, getting to know them. (CoC midwife 2)*

*You may have concerns with the family and Social Care do and then they ring you and as a regular community midwife they say oh how’s this and how’s that and you think well I don’t really know because I’ve seen her for 15/20 minutes in an antenatal where I checked her blood pressure, dipped her urine, asked her if everything was alright in the hope that it was and sent her on her way (CoC Midwife 5)*

#### Team structure and flexible working

The establishment of autonomous and flexible working patterns in the CoC team also appeared to facilitate the delivery of the CoC model, as midwives were able to plan and adjust their schedules according to women’s needs. In addition, midwives felt empowered to provide additional appointments where required and visit women at home with full professional freedom without the fear of being questioned.

*And the flexibility of managing my own workload I really, really like, so if I know that a woman needs extra appointments to talk about something, then I’ll book her a double appointment or I’ll book her an appointment the next week or I’ll go see her at home. Being able to go see her at home is, and not having to do that under the radar, is really nice, you know. (CoC midwife, 4)*

*I had a woman when we first started who rang up she was really worried about her screening result for downs and I went off to see her and I was still there at six o’clock and I can remember driving away from it thinking well I wouldn’t have been doing that six months ago. I mean I’d obviously, you know, when I was a regular community midwife if there was a home birth, emergency or something like you’d stay as long as you need but you know, you’re driving away and reflecting thinking now would I really have offered to go and see somebody at five o’clock to talk about a down syndrome screening result? Probably not. I shall come tomorrow, you know, which is a long time if you’re sat at home as a woman worrying about some results. (CoC midwife, 5)*

In contrast, there was a sense of a lack of stability within standard approach teams where midwives frequently rotated through different midwifery settings. This appeared to be coupled with high levels of sickness and last minute staff changes to clinics that all compromised midwives ability to provide continuity of carer to women and build meaningful relationships.

*The teams out here now are not stable, they have to cover sickness, move in and out of hospital and, you know, if we have to cover the hospital, the five that come out have to rotate in and out (Standard approach midwife, 9)*

Communication amongst team members and close working relationships with their associated buddies also promoted a sense of team cohesion and support amongst CoC midwives. Moreover, CoC team were all employed for the duration of the pilot and were not required to rotate as part of usual midwifery practice.

*So I think because we do work with the smaller caseloads, it's much easier for everyone to be more involved in each other's ladies and know what's going on with any particular cases of concern, and what's going on personally as well with your colleagues, so you know, not just work-wise but your outside factors, what's going on in your personal life, if they want to share that of course and they might not want to do, but it's easier to keep a track on that and know what's happening and be more supportive for your work as well as what's going on personally as well. (CoC midwife 1)*

## Discussion

This is the first qualitative evaluation of the implementation of a CoC model in the UK that prioritised continuity for women’s antenatal and postnatal care, excluding birth. Implementation successes were largely due to additional resources including time, reduced caseloads for midwives, fixed team structure and autonomous diary management. High job satisfaction amongst CoC team midwives was underpinned by perceptions of providing high quality personalised care to women, good working relationships with their individual buddies as well as the whole team.

Our evaluation also included the views of midwives and women from standard approach teams within the same geographical area. Whilst there were many similarities in the characteristics and needs of women seen by the CoC and standard approach teams, key resource differences between the two models appeared to impact both women’s satisfaction with their care as well as midwives’ job satisfaction and sense of role fulfilment. A contributing factor to the positive job perceptions of CoC midwives over standard approach midwives may be that the CoC midwives self-selected for the role of CoC (i.e. they applied for the position), whereas many of the standard approach midwives were rotational midwives allocated their community role for a specified period of time. Standard approach midwives reported increased time pressures conflicting with large caseloads, lack of consistent administrative and maternity support staff, low staffing levels, being on-call and covering sickness and annual leave across for other community midwifery teams and rotational working patterns; all contributing to midwives’ feelings of stress and burnout as well as impacting their ability to provide continuity and personalised care. These feelings were echoed by women receiving care through the standard approach where many reported feeling rushed and unable to discuss key concerns, especially surrounding mental health, as they did not want to add to the burden of their midwife.

The model implemented differs to the model of full CoC that advocates women seeing see the same midwife throughout pregnancy, birth and postnatally. Although there is a wealth of positive evidence for birth associated outcomes and women’s satisfaction with this model of care [1], midwives’ experiences have varied and previous applications of the full CoC model have been short-lived [20, 21]. Nevertheless full CoC is widely advocated as the gold standard for care through the ‘Better Births’ and national maternity transformation policy initiatives [3]. A key challenge is how maternity providers can implement and scale up delivery of the CoC model that includes birth in a feasible and sustainable way in the face of NHS budget constraints and a national shortage of midwives [22]. Further, it is not entirely clear which aspects of the CoC model determine the positive outcomes outlined for women and babies; is it the self-selection by midwives, the relational aspects human factor or other structural aspects of the care model such as team size, and reduced time constraints or ability to provide care in different settings such as home visits? The likelihood is that it is a combination of all these factors.

Previous studies have found autonomous and flexible practice to be key components of the full CoC model [6, 8, 23]. In our evaluation, we found these aspects also facilitated delivery of the CoC model where additional time and the reduced numbers of women were integral to successful implementation. However, the experiences of midwives working within a standard approach highlighted that teams serving a similar population of women with increasing vulnerabilities and psycho-social complexities are potentially working in very different and inequitable models of care. The high levels of stress and burnout reported by standard approach midwives is consistent with other studies on the general well-being of midwives as well as studies that include continuity at birth [20]. A recent cross-sectional survey with UK midwives found many were unable and/or unwilling to work within a CoC model including birth due to the requirement of being on-call for birth [10]. Barriers included caring responsibilities, concerns about quality and safety of cross organisational working and work-life balance [10]. Burnout in healthcare staff has been associated with increased errors, poor quality of care and reduced patient satisfaction [24].

Midwives aim to provide high quality and consistent care to all women. When they are prevented from doing this for whatever reason, they are likely to feel dissatisfied. Our findings suggest that burnout in midwives may also be linked to a perceived lack of role fulfilment and not being able to provide a standard of care midwives were satisfied with due to resource constraints. Alongside women, midwives are at the heart of the CoC philosophy, yet the UK NHS has difficulties achieving recommended midwifery staffing levels and this hinders the implementation and sustainability of the full CoC model [25]. Prioritisation of continuity of carer for antenatal and postnatal care in the model may offer an alternative systems approach that balances the key resources enabling sustainable models of care and still provides positive outcomes for women and babies and those caring for them. Analysis of birth outcome data for this type of CoC model is greatly needed to determine whether this model of care can help improve birth outcomes.

A consistent and repeated benefit reported by women and midwives of the extended time and personalised focus of the CoC team was in relation to the disclosure of mental health issues. Every appointment with CoC team appeared to focus on women’s well-being including mental health. In contrast, women receiving care from the standard approach team said they withheld discussing their concerns as they could see their midwives were stressed and did not want to add to their burden. Women’s mental health during pregnancy and after birth is known to influence the emotional health and well-being of their children [26, 27]. However, there are few services available across the country focussed on supporting women with mild to moderate mental difficulties [28]. The recent national perinatal maternal morbidity review found suicide to be the third largest cause of direct maternal deaths occurring during, or within the 42 days of the end of pregnancy, and is the leading cause of deaths occurring within the first year after birth [29]. There is evidence that mental health difficulties in the perinatal period are poorly understood and underreported and there is an urgent need to ensure women receive appropriate and timely support to prevent exacerbation of difficulties [30].

In our study, both CoC and standard approach midwives cited language barriers, increasing safeguarding concerns and other social difficulties amongst the key challenges of delivering personalised care to a diverse group of women. Recent evidence suggests women from ethnic minority backgrounds are up to five times more likely to die during pregnancy than White British women [29] and overall, have less positive experiences of maternity care [31]. Midwives are often the only health professional some women may engage with during pregnancy yet there is very little time in the standard care delivery approach to discuss women’s concerns in detail. Women from ethnic minority backgrounds and those living in areas of high deprivation often experience multiple morbidities and disadvantages and as such, are likely to benefit from more directly accessible maternity care including longer appointments and increased flexibility of visits as in the CoC model. Indeed, CoC midwives shared many examples of identification of domestic violence and mental health difficulties following a period of building up trusting relationships with women and visiting them at home (e.g. for the 36 week birth plan home visit). Further research should therefore focus on identifying effective implementation strategies to help scale up continuity of carer models that not only work for women experiencing multiple disadvantages, but also enable midwives to provide high quality, personalised care to women.

### Strengths and limitations

To our knowledge, this is the first in-depth evaluation of a CoC model that prioritises antenatal and postnatal continuity. Our focus on implementation highlighted many key components that aided the successful delivery of the model. Aside from postnatal continuity, few challenges to implementing the model were reported, possibly alluding to a positivity bias in the accounts of women who were already part of our BiBBS cohort study [16] and midwives as they were aware of the evaluation aims. Moreover, the CoC model benefited from a highly valuable and protected source of funding where staff were exclusively employed to work in the CoC team and on-calls and sickness cover were not shared with standard approach midwives. Therefore, these findings may not be directly generalisable to all settings, particularly where CoC models are being implemented within existing maternity funding structures. Whilst choice is a key consideration for women, it is important to acknowledge that midwives in different life stages and those with caring responsibilities may have differing preferences for CoC working and on-calls which has implications for future recruitment of midwives to CoC models. Future research may benefit from taking a longitudinal approach to further understand the challenges and barriers to implementing within CoC models being applied across different settings.

## Conclusion

The CoC model was an acceptable model of care for women and midwives, despite missing the intrapartum element typically included in other models of its kind. The combination of additional time and reduced volume of women alongside other structural resource strengths aided the successful implementation of the model. Although maternity providers across the UK are striving towards providing full CoC to women, this level of continuity can be hard to maintain in a system with staff shortages and an often depleting workforce, combined with the need to be on-call for births. Our model may represent a more feasible and sustainable option to implement; however, there is still a need to ensure that care at birth is more integrated to ensure all women receive continuity in the quality of care throughout their maternity journey. High quality care is not just good communication but also requires consistency and equity in the care and support available to women and, the balancing of workload and key resources, including support staff, across the maternity system.

## Supplementary Information

**Additional file 1.**

**Additional file 2.**

**Additional file 3.**

## Data Availability

The data that support the findings of this study are available on request from the corresponding author. The data are not publicly available due to privacy or ethical restrictions.

## Abbreviations

BiB: Born in Bradford
BiBBS: Born in Bradford’s Better Start
CoC: Continuity of Carer
FGM: Female genital mutilation
HRA: Health Research Authority
MRC: Medical Research Council
NHS: National Health Service
TA: Thematic Analysis
TDF: Theoretical Domains Framework
