# Supplementary material for: The key components of a successful model of midwifery-led continuity of carer, without continuity at birth: findings from a qualitative implementation evaluation
Source: BMC Pregnancy Childbirth. 2021 Mar 12;21:205. doi: 10.1186/s12884-021-03671-2 (PMC7955626; doi:10.1186/s12884-021-03671-2)
Supplement: Supplementary file 1 — Additional file 1. [file 12884_2021_3671_MOESM1_ESM.docx]

**CoC Team Midwives Interview Topic Guide**

**Background information**

1. *To start off I would like to ask a few questions about your midwifery career to date:*
   1. *How many years have you been practising as a qualified midwife?*
   2. *When did you start working in the OPAL team?*
   3. *What did you do prior to gaining this position?*
   4. (If not answered in question D) *Have you worked with women in this area before?*

**Working in the Personalised Midwifery model**

*I am now going to ask some questions about the Personalised Midwifery model:*

1. Can you tell me about the Personalised Midwifery model and how the model works?

*Prompt where necessary – e.g. what was a typical day like? (Knowledge)*

1. How is the personalised midwifery model different to the standard model of care that women receive in this area? *If not answered above (Professional role)*
2. How big is the caseload you have been looking after? (*Professional role; Fidelity*)
3. Were any changes made to the model compared to the way it was planned? If so, what changes were made*? (Environmental context and resources; Fidelity)*
4. What were your expectations of working in this model prior to joining the team? *Prompt: Were your expectations met? (Beliefs about consequences)*
5. What do you think are the benefits of this kind of personalised midwifery model? For midwives? For women? *(Beliefs about consequences)*
6. What have been the challenges or drawbacks of working the personalised model? For midwives? For women? (*Beliefs about consequences)*
7. What support strategies could help with those challenges? *Prompt where necessary; were these challenges overcome? Were any challenges not resolved?* *(Beliefs about capabilities)*
8. How satisfied are you working in the Personalised Midwifery model? How does that compare to how satisfied you were when working in a standard care model? *(Emotions)*
9. Which aspects of the role/model do you enjoy? And why? *(Emotions)*
10. What aspects of the role/model do you not enjoy as much? And why? *(Emotions)*
11. Are there any support strategies or resources that that could help improve your job satisfaction? (*environmental context and resources*)
12. From your experience, what further changes would be required in order for the personalised midwifery model to be rolled out successfully? *(Environmental context and resources*)
13. How supportive are other midwives/health professionals of the personalised midwifery model *(social influence)*

**Familiarity, reflections and challenges on working with the local population**

1. How familiar were you with the local population prior to joining the team? What have been the main support needs of women? *(Knowledge*)
2. What have been the challenges in providing personalised midwifery care to women in this area? (*environmental context and resources*)
3. What has been the emotional impact on you as a health professional caring for these women? *(Emotions)*
4. What has been the impact on your professional confidence *(beliefs about capabilities)*
5. From your experience, how well are the needs of women you care for met by local services? E.g. for mental health (*environmental context and resources)*
6. What have been the main barriers you have encountered when accessing support for women? What barriers have women faced? (*environmental context and resources)*
7. How have these barriers been overcome? What could be done to help overcome any outstanding barriers?

*(environmental context and resources)*

**Ending the interview**

1. Is there anything else you’d like to say or anything you thought you wanted to discuss before the interview that we have not talked about today?
2. What would you like to see coming out of the study?
3. What is the one message you’d like to give commissioners about the personalised midwifery model?
4. Do you have any questions for me?

***Thank you very much for taking part in the interview today***
