# Supplementary material for: The key components of a successful model of midwifery-led continuity of carer, without continuity at birth: findings from a qualitative implementation evaluation
Source: BMC Pregnancy Childbirth. 2021 Mar 12;21:205. doi: 10.1186/s12884-021-03671-2 (PMC7955626; doi:10.1186/s12884-021-03671-2)
Supplement: Supplementary file 2 — Additional file 2. [file 12884_2021_3671_MOESM2_ESM.doc]

**Standard care Midwives Interview Topic Guide**

**Background information**

1. *To start off I would like to ask a few questions about your midwifery career to date:*
   1. *How many years have you been practising as a qualified midwife?*
   2. *When did you start working in the community? How long in Bradford?*
   3. *Have you worked with women in this area before?*
   4. *What did you do prior to gaining this position?*

**Working in the Standard Care Midwifery model**

*I am now going to ask some questions about the current midwifery model:*

1. Can you tell me about how the current community midwifery care model works?

*Prompt where necessary – e.g. what is a typical day like? (Knowledge)*

1. How big is the caseload you have been looking after? (*Professional role; Fidelity*)
2. What do you think are the benefits of this kind of midwifery model? For midwives? For women? *(Beliefs about consequences)*
3. What have been the challenges or drawbacks of working in this model? For midwives? For women? (*Beliefs about consequences)*
4. What support strategies could help with those challenges? *Prompt where necessary; were these challenges overcome? Were any challenges not resolved?* *(Beliefs about capabilities)*
5. Which aspects of the role/model do you enjoy? And why? *(Emotions)*
6. What aspects of the role/model do you not enjoy as much? And why? *(Emotions*
7. How satisfied are you working in the current midwifery care model?
8. Are there any support strategies or resources that that could help improve your job satisfaction? (*environmental context and resources*)
9. How important is it for midwives to see the same woman throughout her pregnancy and after birth, and why?
10. And how important is it for women to see the same midwife throughout their pregnancy and after birth, and why?
11. Are you familiar with the personalised caseloading midwifery care model? What are your views on this type of midwifery model?
12. What would be the key aspects you would like to see in a personalised caseloading model if it was to be rolled out successfully in your area? How would it work? What resources and support would be required?
13. What would be the benefits of working in a caseloading model? For midwives? For women?
14. What would be the drawbacks? For midwives? For women?
15. How supportive would other midwives/health professionals you know be of a personalised caseloading midwifery model in Bradford *(social influence)*

**Familiarity, reflections and challenges on working with the local population**

1. How familiar were you with the local population prior to joining the team? What have been the main support needs of women? *(Knowledge*)
2. What have been the challenges in providing midwifery care to women in this area? (*environmental context and resources*)
3. What has been the emotional impact on you as a health professional caring for these women? *(Emotions)*
4. What has been the impact on your professional confidence *(beliefs about capabilities)*
5. From your experience, how well are the needs of women you care for met by local services? E.g. for mental health (*environmental context and resources)*
6. What have been the main barriers you have encountered when accessing support for women? What barriers have women faced? (*environmental context and resources)*
7. How have these barriers been overcome? What could be done to help overcome any outstanding barriers? *(environmental context and resources)*

**Ending the interview**

1. Is there anything else you’d like to say or anything you thought you wanted to discuss before the interview that we have not talked about today?
2. What would you like to see coming out of the study?
3. What is the one message you’d like to give commissioners about the way midwifery care is delivered?
4. Do you have any questions for me?

***Thank you very much for taking part in the interview today***
