# Supplementary material for: The key components of a successful model of midwifery-led continuity of carer, without continuity at birth: findings from a qualitative implementation evaluation
Source: BMC Pregnancy Childbirth. 2021 Mar 12;21:205. doi: 10.1186/s12884-021-03671-2 (PMC7955626; doi:10.1186/s12884-021-03671-2)
Supplement: Supplementary file 3 — Additional file 3. [file 12884_2021_3671_MOESM3_ESM.doc]

**Women’s Topic Guide**

1. To begin with, I’d like to ask you a few background questions about yourself.

Is this your first baby or do you have any other children?

How old are they? Were they all born in Bradford?

*For mothers with other children*

1. Thinking back to when you had your last baby, what was different about the care you received from your midwives this time? Which aspects were the same?

*Check participants understand who was a midwife to distinguish from other health professionals e.g health visitor*

1. How did you find the care you received from your midwives in pregnancy?

Prompt: which aspects did you find helpful?

Which aspects of your care did you enjoy?

Which aspects could be improved?

*Additional prompt if required on type of care: e.g the way they looked after you, the information or support they gave*

1. How did you find the care you received at home from your midwives after your baby was born?

Prompt: which aspects did you find helpful?

Which aspects of your care were good?

Which aspects could be improved?

*Additional prompt if required on type of care: e.g the way they looked after you, the information or support they gave*

1. How many midwives did you see during pregnancy?

How often did you see the same midwife? How long were your appointments?

Did you feel that was enough? Why or why not?

1. How many midwives did you see after you had your baby? How often did you see the same midwife? How long were your appointments?

Did you feel that was enough? Why or why not?

1. What are your thoughts on seeing the same midwife throughout pregnancy and after you have had your baby?

Prompt: do you think it makes a difference or not?

Why?

What were/could be the positive aspects?

What were/could be the negative aspects?

1. How do you think midwives find seeing the same women throughout pregnancy and after birth?

What could be the positive aspects?

What could be the negative aspects?

1. For you, how important was it to see the same midwife all the way through your pregnancy?
2. And how important was it for you to see the same midwife after you had your baby?
3. What was your relationship like with your midwife?

Prompt: which aspects were good about it, and why? Which aspects were difficult, and why?

Did you trust your midwife?

1. How involved did you feel in decisions about your pregnancy?
2. How involved did you feel in decisions about planning the birth?
3. How involved did you feel in decisions after your baby was born?
4. How important was it for you to feel involved in the decisions about your pregnancy, the birth and after you had your baby?
5. How easy or difficult was it for you to talk to your midwife about your feelings? Can you give me an example?

Prompt: Did it make a difference which midwife was there to speak to?

1. How easy or difficult was it for you to talk to your midwife about any advice or support that you wanted? Can you give me an example?

Prompt: Did it make a difference which midwife was there to speak to?

1. Did you feel you got the advice and support that you wanted from your midwife? Prompt: what would’ve been helpful to get the support that you needed? What further information did you need?
2. Overall, how satisfied are you with the care you received from the midwives? Why is that?
3. Are there any aspects that could be changed that would increase your satisfaction? What, how and why?

*For women who needed an interpreter*

1. How often did you have the same interpreter in your appointments?
2. Did it matter if the same interpreter was there for all your appointments?

What were/could be the positive aspects? What were/could be the negative aspects?

1. How comfortable were you in talking to your midwife when the interpreter was there?

Prompt: Was the interpreter someone you knew or from your community?

How well did you understand the information shared?

*Engagement with BSB projects*

1. Have you heard about Better Start Bradford?

If yes, what is your understanding of BSB?

*If no, interviewer to explain BSB at the end of the interview*

1. Were you (or your partner) told about any of the Better Start Bradford projects e.g. welcome to the world, ESOL+, family action perinatal support?
2. Did you go to any pregnancy or baby related groups or courses whilst you were pregnant? Prompt: If so, which ones, where were they held, how did you find them?

If not, why not? Would you have gone to a group if you had known about it? Were you (or your partner) provided information any information about events, fun days, open days, any activities taking place in your local community?

1. Have you received any books from the Dolly Parton Imagination Library?

Have you had chance to read them to your baby? How have you found the books?

**Wrapping up**

1. Is there anything else you’d like to say or anything you thought you wanted to discuss before the interview that we have not talked about today?
2. If there was one message you wanted to tell the people who organise midwifery care, what would that be?
3. Do you have any questions for me?

***Thank you very much for taking part in the interview today***
